# Supplementary material for: Decapping factor Dcp2 controls mRNA abundance and translation to adjust metabolism and filamentation to nutrient availability
Source: eLife. 2023 Jun 2;12:e85545. doi: 10.7554/eLife.85545 (PMC10287164; doi:10.7554/eLife.85545)
Supplement: Figure 7—source data 1. — Each panel lists the MIPS functional categories enriched among the genes encoding each group of transcripts, the p-value indicating the statistical significance of enrichment, the number of genes represented by the mRNAs in the set belonging to the functional category (k), and the total number of genes present in the functional group (f). The Bonferroni correction and a minimum p-value of 0.05 were applied. [file elife-85545-fig7-data1.zip › Figure 7 - source data 1.docx]

1. **GO analysis of mRNA_up_*dcp2*△ (n=1376)**

| **Functional category** | **P-value** | **k** | **f** |
| --- | --- | --- | --- |
| metabolism of energy reserves | 6 x 10^-8^ | 30 | 56 |
| sugar, glucoside, polyol and  carboxylate catabolism | 4 x 10^-7^ | 37 | 81 |
| tricarboxylic-acid pathway | 1 x 10^-6^ | 19 | 31 |
| c-compound and carbohydrate transport | 1 x 10^-6^ | 20 | 34 |
| c-compound and carbohydrate metabolism | 8 x 10^-6^ | 74 | 223 |
| stress response | 1 x 10^-5^ | 57 | 162 |
| meiotic recombination | 1 x 10^-5^ | 20 | 38 |

1. **GO analysis of mRNA_dn_*dcp2*△ (n=1281)**

| **Functional category** | **P-value** | **k** | **f** |
| --- | --- | --- | --- |
| ribosomal proteins | < 10^-14^ | 153 | 246 |
| rRNA processing | < 10^-14^ | 90 | 169 |
| RNA binding | 1 X 10^-14^ | 82 | 189 |
| ribosome biogenesis | 1 X 10^-12^ | 38 | 64 |
| translation initiation | 4 X 10^-11^ | 27 | 40 |
| N-directed glycosylation, deglycosylation | 6 X 10^-7^ | 23 | 43 |
| unfolded protein response | 1 X 10^-6^ | 31 | 69 |
| protein folding and stabilization | 1 X 10^-6^ | 38 | 93 |
| rRNA synthesis | 2 X 10^-6^ | 26 | 55 |
| tRNA synthesis | 4 X 10^-6^ | 20 | 38 |
| peptidoglycan anabolism | 6 X 10^-6^ | 12 | 17 |
| non-vesicular ER transport | 8 X 10^-6^ | 11 | 15 |
| rRNA modification | 2 X 10^-5^ | 12 | 18 |
| translation elongation | 2 X 10^-5^ | 13 | 21 |
| aminoacyl-tRNA-synthetases | 3 X 10^-5^ | 19 | 39 |
| tRNA modification | 5 X 10^-5^ | 20 | 43 |
| biosynthesis of methionine | 5 X 10^-5^ | 6 | 6 |

1. **GO analysis of Dhh1-dep. mRNA_up_*dcp2*△ (n=752)**

| **Functional category** | **P-value** | **k** | **f** |
| --- | --- | --- | --- |
| sugar, glucoside, polyol and  carboxylate catabolism | 9 x 10^-12^ | 33 | 81 |
| metabolism of energy reserves | 3 x 10^-10^ | 25 | 56 |
| tricarboxylic-acid pathway | 4 x 10^-9^ | 17 | 31 |
| stress response | 2 x 10^-7^ | 42 | 162 |
| c-compound and carbohydrate metabolism | 2 x 10^-7^ | 52 | 223 |
| sugar, glucoside, polyol and  carboxylate anabolism | 2 x 10^-6^ | 15 | 35 |
| c-compound and carbohydrate transport | 5 x 10^-6^ | 14 | 34 |
| Oxidative stress response | 8 x 10^-5^ | 17 | 55 |
| biosynthesis of glutamate | 8 x 10^-5^ | 8 | 15 |
| regulation of glycolysis and gluconeogenesis | 9 x 10^-5^ | 9 | 19 |

1. **GO analysis of Dhh1-indep. mRNA_up_*dcp2*△ (n=607)**

| **Functional category** | **P-value** | **k** | **f** |
| --- | --- | --- | --- |
| DNA repair | 6 x 10^-9^ | 39 | 159 |
| meiotic recombination | 7 x 10^-8^ | 16 | 38 |
| DNA damage response | 5 x 10^-5^ | 19 | 77 |
| Cell-cell adhesion | 9 x 10^-5^ | 6 | 10 |

1. **GO analysis of TE_up_*dcp2*△ (n=541)**

| **Functional category** | **P-value** | **k** | **f** |
| --- | --- | --- | --- |
| electron transport and membrane-  associated energy conservation | < 10^-14^ | 28 | 58 |
| electron transport | 6 X 10^-13^ | 30 | 83 |
| ribosomal proteins | 3 X 10^-10^ | 51 | 246 |
| energy generation | 7 X 10^-10^ | 13 | 21 |
| mitochondrion | 6 X 10^-9^ | 38 | 170 |
| aerobic respiration | 2 X 10^-8^ | 23 | 77 |
| respiration | 6 X 10^-5^ | 15 | 59 |

1. **GO analysis of TE_dn_*dcp2*△ (n=659)**

| **Functional category** | **P-value** | **k** | **f** |
| --- | --- | --- | --- |
| anion transport | 2 X 10^-5^ | 7 | 11 |
| transcription activation | 3 X 10^-5^ | 14 | 42 |

1. **GO analysis of Ribo_up_*dcp2*△ (n=1261)**

| **Functional category** | **P-value** | **k** | **f** |
| --- | --- | --- | --- |
| aerobic respiration | 8 X 10^-11^ | 40 | 77 |
| sugar, glucoside, polyol and  carboxylate catabolism | 6 X 10^-10^ | 40 | 81 |
| tricarboxylic-acid pathway | 4 X 10^-9^ | 21 | 31 |
| mitochondrion | 6 X 10^-8^ | 62 | 170 |
| metabolism of energy reserves | 7 X 10^-7^ | 27 | 56 |
| oxidative stress response | 2 X 10^-6^ | 26 | 55 |
| electron transport chain and membrane-  associated energy Conservation | 2 X 10^-5^ | 25 | 58 |
| metabolism of vitamins, cofactors, and  prosthetic groups | 4 X 10^-5^ | 20 | 43 |
| biosynthesis of glutamate | 7 X 10^-5^ | 10 | 15 |
| sugar, glucoside, polyol and  carboxylate anabolism | 7 X 10^-5^ | 17 | 35 |

1. **GO analysis of Ribo_dn_*dcp2*△ (n=1326)**

| **Functional category** | **P-value** | **k** | **f** |
| --- | --- | --- | --- |
| ribosomal proteins | < 10^-14^ | 139 | 246 |
| N-directed glycosylation, deglycosylation | 2 X 10^-13^ | 31 | 43 |
| peptidoglycan anabolism | 9 X 10^-7^ | 13 | 17 |
| RNA binding | 1 X 10^-6^ | 66 | 189 |
| O-directed glycosylation, deglycosylation | 1 X 10^-6^ | 12 | 15 |
| rRNA processing | 4 X 10^-6^ | 59 | 169 |
| translation elongation | 5 X 10^-6^ | 14 | 21 |
| ribosome biogenesis | 4 X 10^-5^ | 27 | 64 |
| unfolded protein response | 7 X 10^-5^ | 28 | 69 |
| translation initiation | 9 X 10^-5^ | 19 | 40 |
| sulfate assimilation | 9 X 10^-5^ | 7 | 8 |
