# Supplementary figures and images for: Decapping factor Dcp2 controls mRNA abundance and translation to adjust metabolism and filamentation to nutrient availability
Source: eLife. 2023 Jun 2;12:e85545. doi: 10.7554/eLife.85545 (PMC10287164; doi:10.7554/eLife.85545)

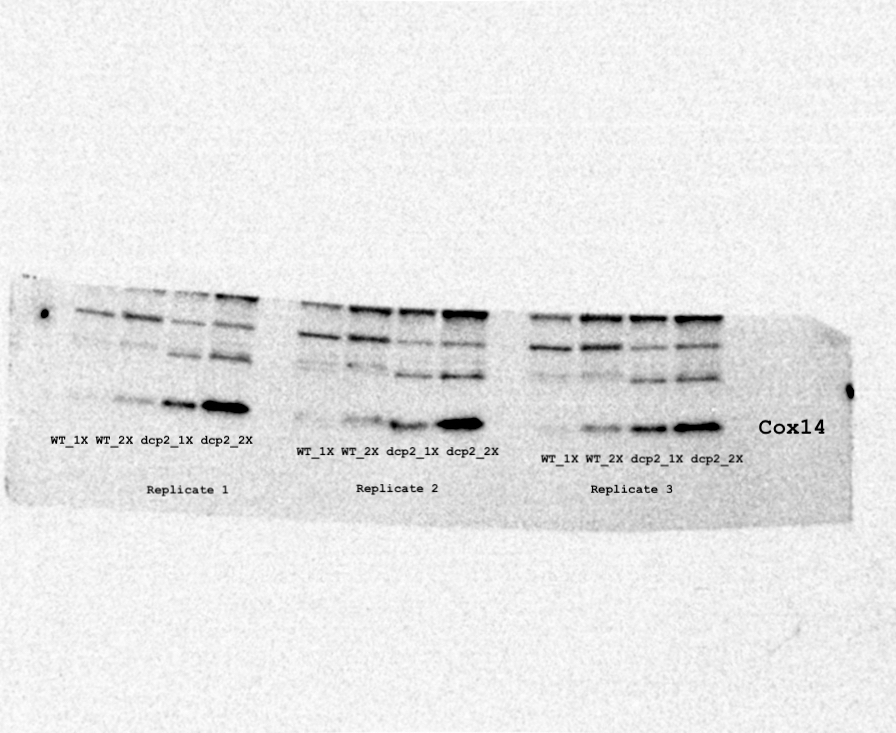

Supplement: Figure 7—source data 2. — Including glucose-repressed (119), NCR (41), metabolism of energy reserves (37), OXPHOS (60), glycosylation (56), sulfur metabolism (16), unfolded protein response (94), agglutinin (16), autophagy-related (26), and ribosomal protein (148) genes, with gene numbers in parenthesis (Figure 7A, B, E, F; Figure 7—figure supplement 1G-H; Figure 7—figure supplement 3A, C, D). [file elife-85545-fig7-data2.zip › Figure 7 - source data 2/Labelled_western-blot_files/Cox14_labelled.jpg]

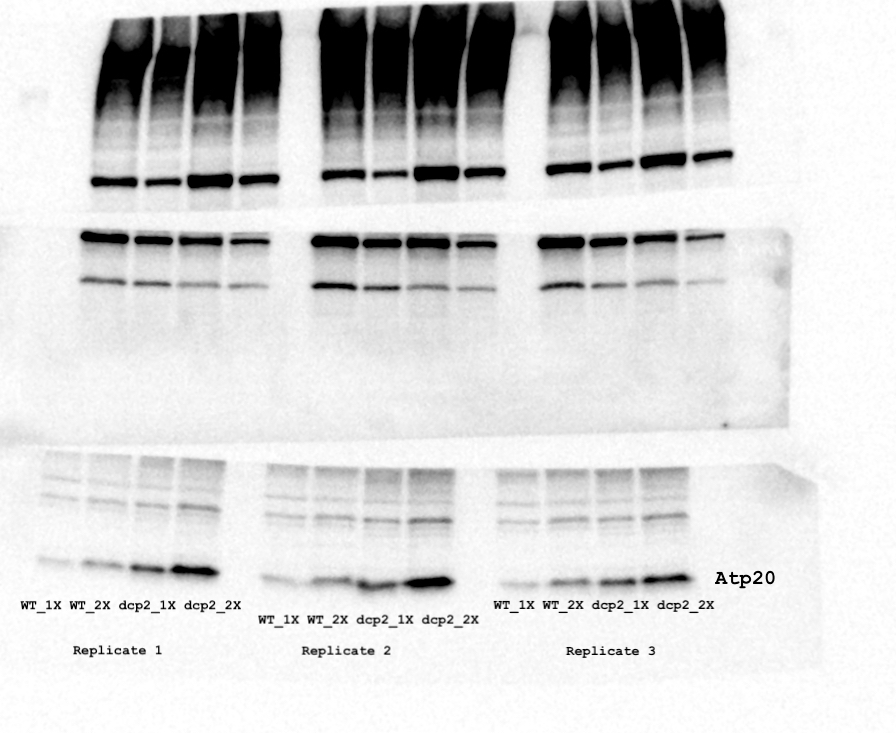

Supplement: Figure 7—source data 2. — Including glucose-repressed (119), NCR (41), metabolism of energy reserves (37), OXPHOS (60), glycosylation (56), sulfur metabolism (16), unfolded protein response (94), agglutinin (16), autophagy-related (26), and ribosomal protein (148) genes, with gene numbers in parenthesis (Figure 7A, B, E, F; Figure 7—figure supplement 1G-H; Figure 7—figure supplement 3A, C, D). [file elife-85545-fig7-data2.zip › Figure 7 - source data 2/Labelled_western-blot_files/Atp20_labelled.jpg]

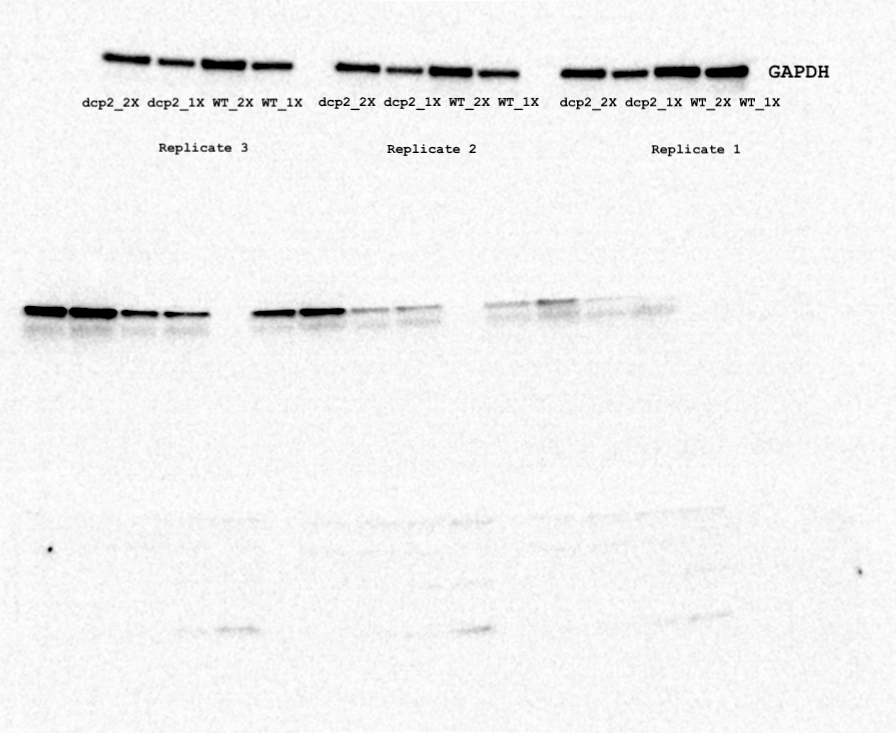

Supplement: Figure 7—source data 2. — Including glucose-repressed (119), NCR (41), metabolism of energy reserves (37), OXPHOS (60), glycosylation (56), sulfur metabolism (16), unfolded protein response (94), agglutinin (16), autophagy-related (26), and ribosomal protein (148) genes, with gene numbers in parenthesis (Figure 7A, B, E, F; Figure 7—figure supplement 1G-H; Figure 7—figure supplement 3A, C, D). [file elife-85545-fig7-data2.zip › Figure 7 - source data 2/Labelled_western-blot_files/GAPDH_labelled.jpg]

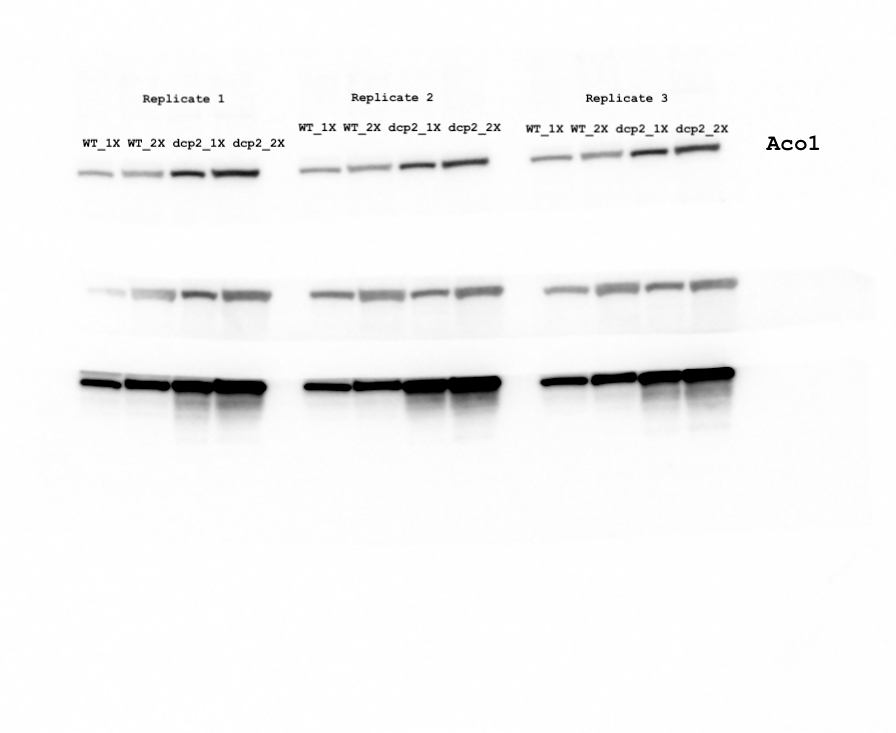

Supplement: Figure 7—source data 2. — Including glucose-repressed (119), NCR (41), metabolism of energy reserves (37), OXPHOS (60), glycosylation (56), sulfur metabolism (16), unfolded protein response (94), agglutinin (16), autophagy-related (26), and ribosomal protein (148) genes, with gene numbers in parenthesis (Figure 7A, B, E, F; Figure 7—figure supplement 1G-H; Figure 7—figure supplement 3A, C, D). [file elife-85545-fig7-data2.zip › Figure 7 - source data 2/Labelled_western-blot_files/Aco1_labelled.jpg]

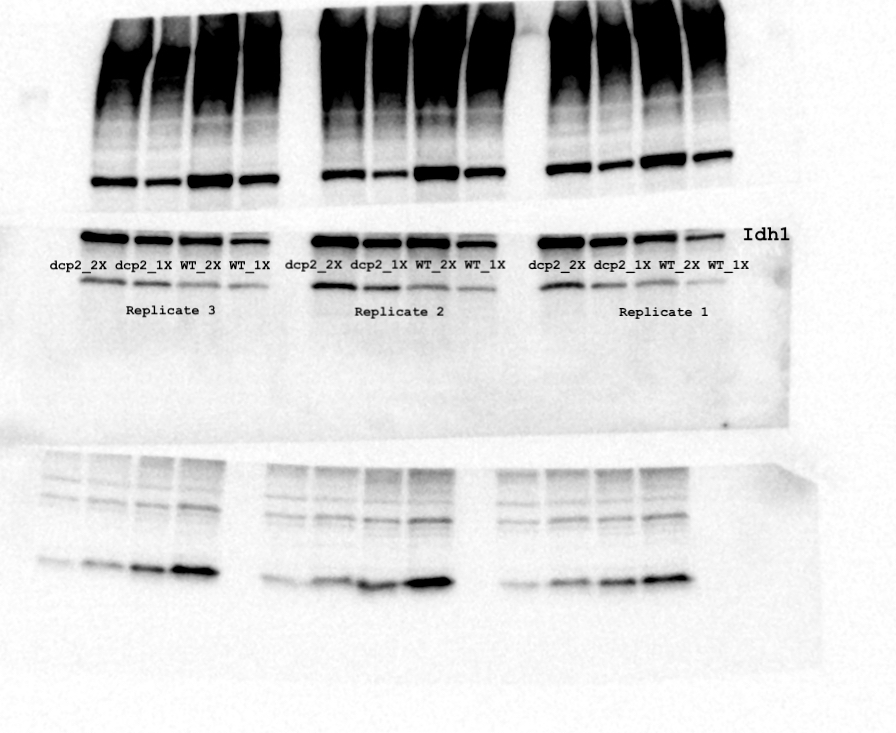

Supplement: Figure 7—source data 2. — Including glucose-repressed (119), NCR (41), metabolism of energy reserves (37), OXPHOS (60), glycosylation (56), sulfur metabolism (16), unfolded protein response (94), agglutinin (16), autophagy-related (26), and ribosomal protein (148) genes, with gene numbers in parenthesis (Figure 7A, B, E, F; Figure 7—figure supplement 1G-H; Figure 7—figure supplement 3A, C, D). [file elife-85545-fig7-data2.zip › Figure 7 - source data 2/Labelled_western-blot_files/Idh1_labelled.jpg]

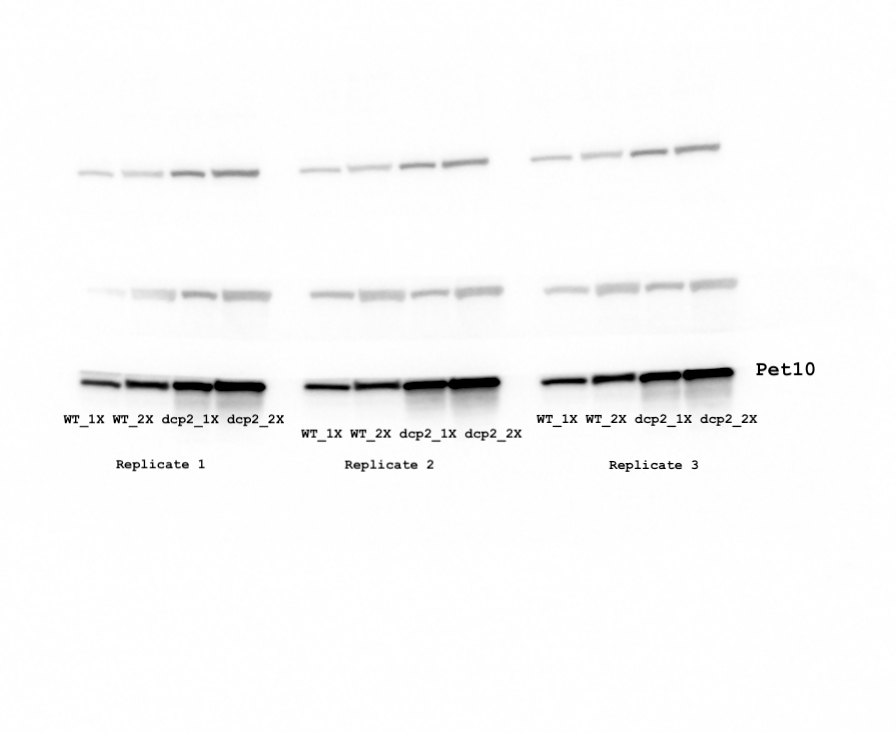

Supplement: Figure 7—source data 2. — Including glucose-repressed (119), NCR (41), metabolism of energy reserves (37), OXPHOS (60), glycosylation (56), sulfur metabolism (16), unfolded protein response (94), agglutinin (16), autophagy-related (26), and ribosomal protein (148) genes, with gene numbers in parenthesis (Figure 7A, B, E, F; Figure 7—figure supplement 1G-H; Figure 7—figure supplement 3A, C, D). [file elife-85545-fig7-data2.zip › Figure 7 - source data 2/Labelled_western-blot_files/Pet10_labelled.jpg]

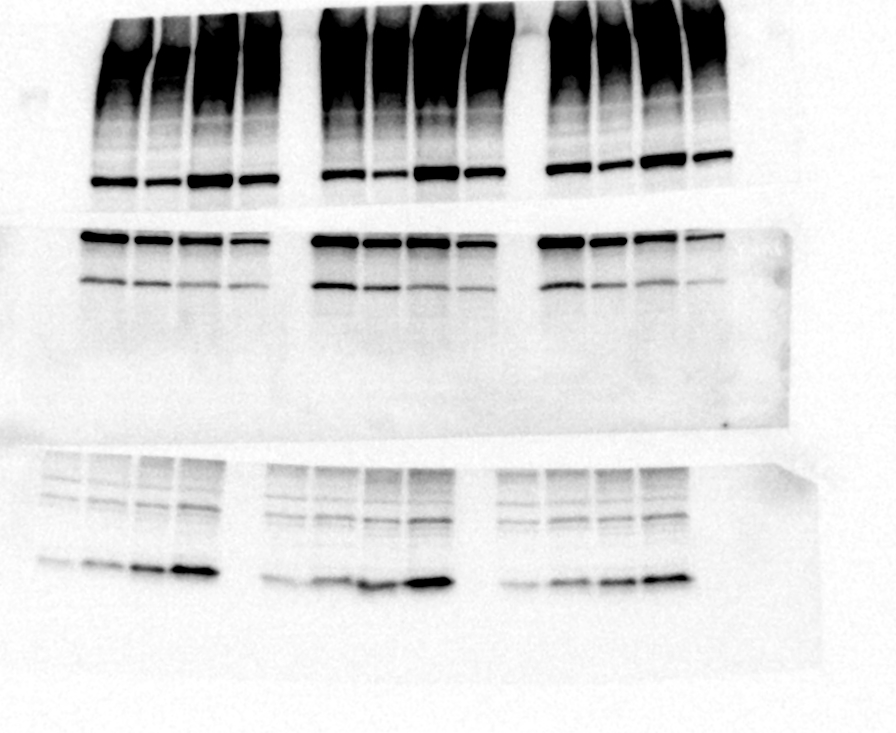

Supplement: Figure 7—source data 2. — Including glucose-repressed (119), NCR (41), metabolism of energy reserves (37), OXPHOS (60), glycosylation (56), sulfur metabolism (16), unfolded protein response (94), agglutinin (16), autophagy-related (26), and ribosomal protein (148) genes, with gene numbers in parenthesis (Figure 7A, B, E, F; Figure 7—figure supplement 1G-H; Figure 7—figure supplement 3A, C, D). [file elife-85545-fig7-data2.zip › Figure 7 - source data 2/Original_western-blot_files/Atp20.jpg]

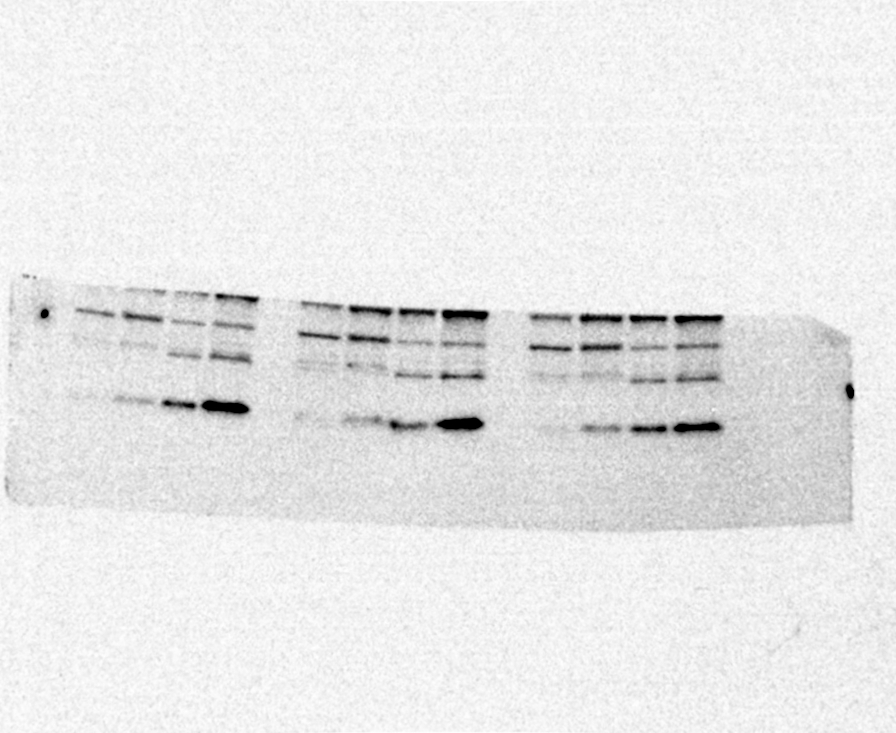

Supplement: Figure 7—source data 2. — Including glucose-repressed (119), NCR (41), metabolism of energy reserves (37), OXPHOS (60), glycosylation (56), sulfur metabolism (16), unfolded protein response (94), agglutinin (16), autophagy-related (26), and ribosomal protein (148) genes, with gene numbers in parenthesis (Figure 7A, B, E, F; Figure 7—figure supplement 1G-H; Figure 7—figure supplement 3A, C, D). [file elife-85545-fig7-data2.zip › Figure 7 - source data 2/Original_western-blot_files/Cox14.jpg]

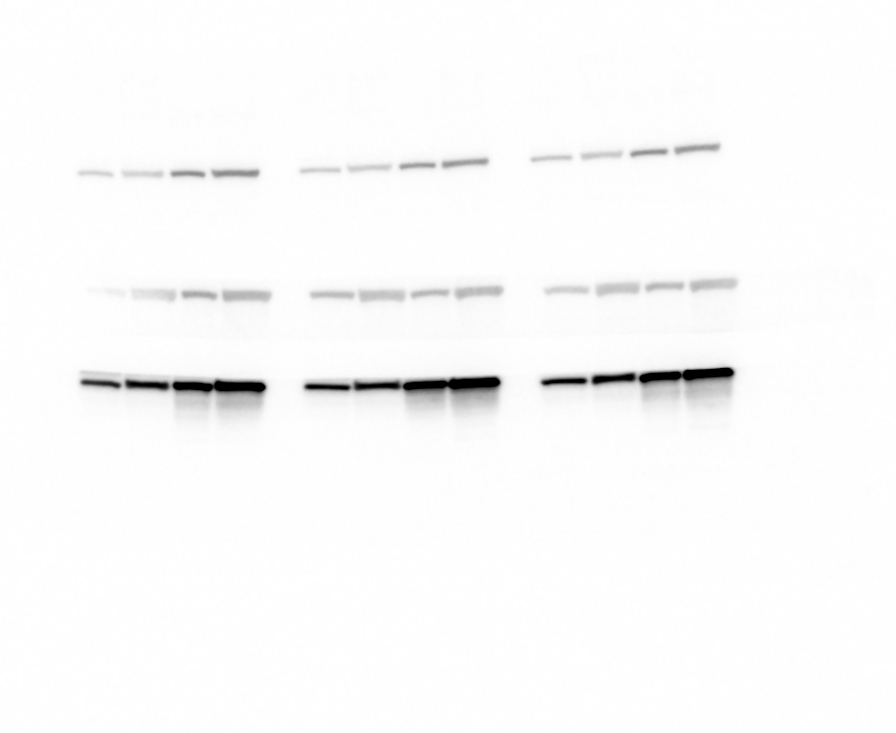

Supplement: Figure 7—source data 2. — Including glucose-repressed (119), NCR (41), metabolism of energy reserves (37), OXPHOS (60), glycosylation (56), sulfur metabolism (16), unfolded protein response (94), agglutinin (16), autophagy-related (26), and ribosomal protein (148) genes, with gene numbers in parenthesis (Figure 7A, B, E, F; Figure 7—figure supplement 1G-H; Figure 7—figure supplement 3A, C, D). [file elife-85545-fig7-data2.zip › Figure 7 - source data 2/Original_western-blot_files/Pet10.jpg]

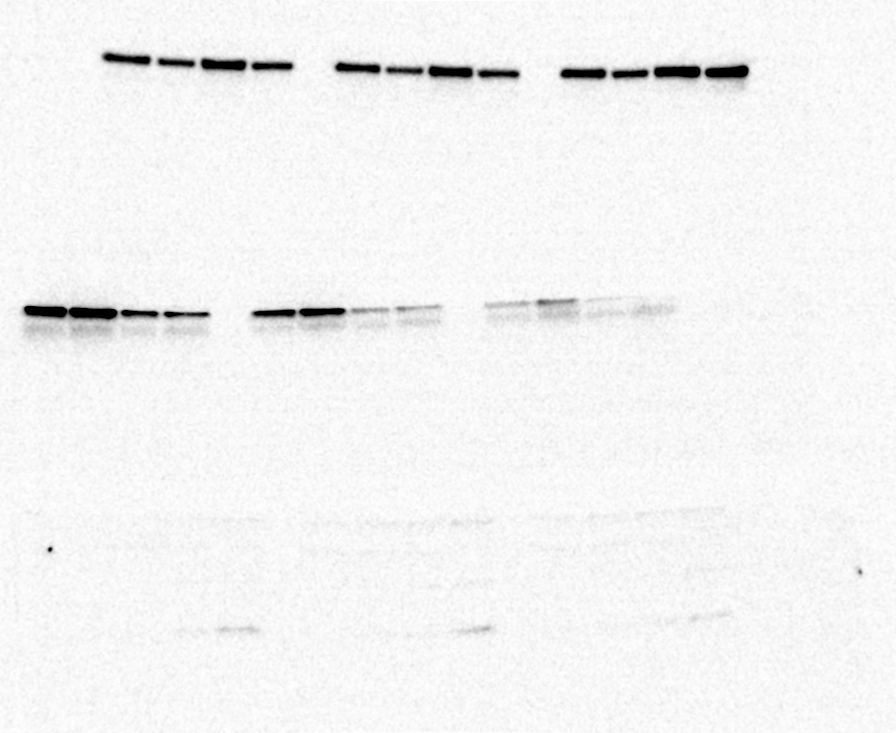

Supplement: Figure 7—source data 2. — Including glucose-repressed (119), NCR (41), metabolism of energy reserves (37), OXPHOS (60), glycosylation (56), sulfur metabolism (16), unfolded protein response (94), agglutinin (16), autophagy-related (26), and ribosomal protein (148) genes, with gene numbers in parenthesis (Figure 7A, B, E, F; Figure 7—figure supplement 1G-H; Figure 7—figure supplement 3A, C, D). [file elife-85545-fig7-data2.zip › Figure 7 - source data 2/Original_western-blot_files/GAPDH.jpg]

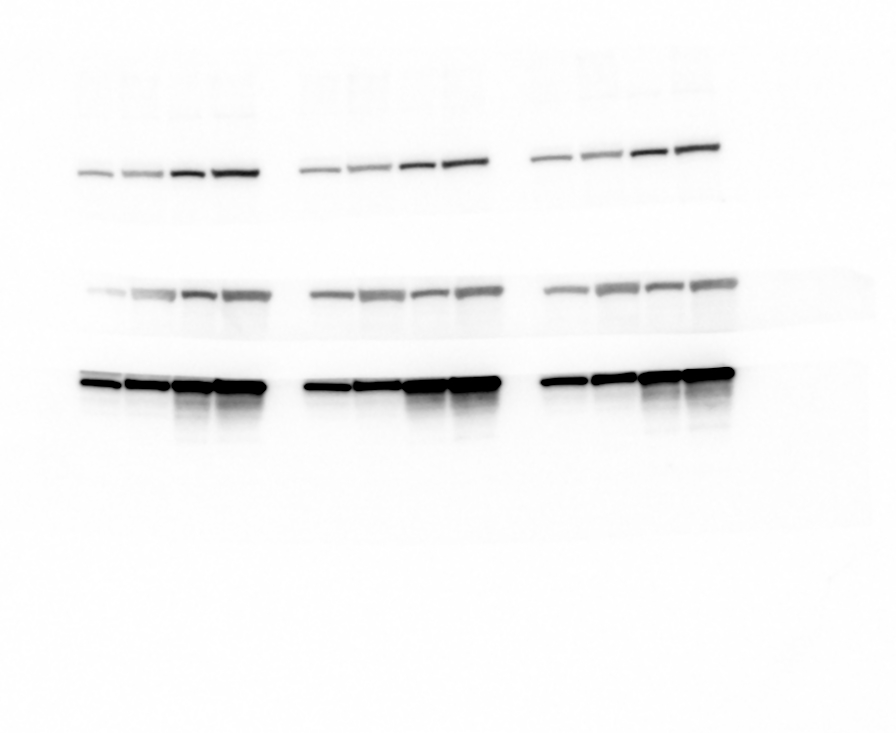

Supplement: Figure 7—source data 2. — Including glucose-repressed (119), NCR (41), metabolism of energy reserves (37), OXPHOS (60), glycosylation (56), sulfur metabolism (16), unfolded protein response (94), agglutinin (16), autophagy-related (26), and ribosomal protein (148) genes, with gene numbers in parenthesis (Figure 7A, B, E, F; Figure 7—figure supplement 1G-H; Figure 7—figure supplement 3A, C, D). [file elife-85545-fig7-data2.zip › Figure 7 - source data 2/Original_western-blot_files/Aco1.jpg]
