# Supplementary material for: Decapping factor Dcp2 controls mRNA abundance and translation to adjust metabolism and filamentation to nutrient availability
Source: eLife. 2023 Jun 2;12:e85545. doi: 10.7554/eLife.85545 (PMC10287164; doi:10.7554/eLife.85545)
Supplement: Supplementary file 1. — File 1d contains details of the nLUC reporter genes. File 1d contains the nLUC CDS codon optimized for S. cerevisiae. [file elife-85545-supp1.docx]

**Supplementary File 1**

**Supplementary File 1a: Yeast strains used in this study**

| Strain | Genotype | Source |
| --- | --- | --- |
| F2147/W303 (WT) | *MATa ade2-1 ura3-1 his3-11,15 trp1-1 leu2-3,112 can1-100* | (He et al. 2003) |
| CFY1016 (*dcp2*∆) | *MATa ade2-1 ura3-1 his3-11,15 trp1-1 leu2-3,112 can1-100 dcp2::HIS3* | (He et al. 2003) |
| H5217 (*dhh1*Δ) | *MATa ade2-1 ura3-1 his3-11,15 trp1-1 leu2-3,112 can1-100 dhh1*∆*::kanMX4* | (Zeidan et al. 2018) |
| H5307 (*dhh1*Δ*dcp2*∆) | *MATa ade2-1 ura3-1 his3-11,15 trp1-1 leu2-3,112 can1-100 dcp2*Δ*::HIS3 dhh1*∆*::kanMX4* | (Zeidan et al. 2018) |
| HFY1081 (*xrn1*Δ) | *MATa ade2-1 ura3-1 his3-11,15 trp1-1 leu2-3,112 can1-100 xrn1*∆*::ADE2* | (He and Jacobson 2001) |
| BSY3037 (*pat1*Δ) | *MATa ura3-1 trp1-1 ade2-1 leu2-3,112 his3-11,15 pat1*∆*::HIS3* | (Charenton et al. 2017) |
| YFW168 (*pat1*Δ*dhh1*Δ) | *MATa ura3-1 trp1-1 ade2-1 leu2-3,112 his3-11,15 pat1*∆*::HIS3 dhh1*∆*::KanR* | (Charenton et al. 2017) |
| AVK022 (*dcp2-EE*) | *MATa ade2-1 ura3-1 his3-11,15 trp1-1 leu2-3,112 can1-100 dcp2-E149Q,E153Q* | This study |

**Supplementary File 1b: Plasmids used in this study**

| Plasmid | Relevant description | Source or reference |
| --- | --- | --- |
| pQZ145 | *DCP2* in pRS316 | (Zeidan et al. 2018) |
| pAV008 | *dcp2-E149Q,E153Q* in pRS316 | This study |
| YIplac211 | Integrating *URA3* vector | (Gietz and Sugino 1988) |
| pAV013 | *dcp2-E149Q,E153Q* in YIplac211 | This study |
| pRS316 | *CEN6,* *URA3* | (Sikorski and Hieter 1989) |
| pEXfz17-2 | *FLO5-nLUC* in pRS316 | This study |
| pEXfz26-3 | *FIG2-nLUC* in pRS316 | This study |
| pEXfz21-1 | *AAD3-nLUC* in pRS316 | This study |
| pEXfz18-5 | *FLO1-nLUC* in pRS316 | This study |
| pEXfz8-4 | *AMD2-nLUC* in pRS316 | This study |
| pEXfz13-1 | *SAG1-nLUC* in pRS316 | This study |
| pEXfz4-2 | *IDP2-nLUC* in pRS316 | This study |
| pEXfz9-1 | *PCK1-nLUC* in pRS316 | This study |
| pEXfz14-1 | *SSA3-nLUC* in pRS316 | This study |
| pEXfz23-3 | *ADR1-nLUC* in pRS316 | This study |
| pEXfz6-2 | *THI21-nLUC* in pRS316 | This study |
| pEXfz1-2 | *TRK2-nLUC* in pRS316 | This study |
| pEXfz19-1 | *SNZ1-nLUC* in pRS316 | This study |
| pNG158 | *PAT1* in pRS315 | This study |

**Supplementary File 1c: Primers used in this study**

| Primer | Sequence 5’ to 3’ |
| --- | --- |
| AKV005 | TGCATTAGACAAGTGAAAGAACAAATTGGTTTCGATTTGACGG |
| AKV006 | CCGTCAAATCGAAACCAATTTGTTCTTTCACTTGTCTAATGCA |
| AKV010 | GATTAAATCATTTGCTCAAT |
| AKV015 | GCAACTCTCTACCGGGATCA |
| AKV088 | GAAGAAGGATCCGAATCAAAACACAATACAA |
| AKV090 | GAAGAAGAGCTCTCACTTCCTATGCAAAATGC |
| Luc1FP | GTGTTGGGCGCGTTATTTATC |
| Luc1RP | TAGGCTGCGAAATGTTCATACT |
| FPYOR178C | AAAAACGCGGAGCCAAGTTC |
| RPYOR178C | AAAGCTGGACGTCATCAGCA |
| FPYHL024W | CGGTGGCAGCAACTTTAACC |
| FPYHL024W | TTTCGCTTTCCTGCTGGGAT |
| FPYJL153C | AGGCGAAGATGTCCTTGGTG |
| RPYJL153C | GCAGATGGGTCCACTTACCC |
| FPYBL043W | CGCTCGATGCTGTGAGTTTG |
| RPYBL043W | ACCATCGTCGACATCGCTAC |
| FPYGL189C | GAGCTCCACCTCAAAGACCA |
| RPYGL189C | TAAAGCCTTCTTGGCGGCAT |
| FPYMR116C | TCACCATCATTTCTGCCGGT |
| RPYMR116C | TGAATGGCATACACAAATCCACT |
| FPYJL130C | GCTTTCCCAGGTGGCCTATT |
| RPYJL130C | CCGATGGACAAACCACCAGA |
| FPYNR062C | AGCGGCTGGTATCTTCATGG |
| RPYNR062C | GGCAAATGTCGGTGCATTGT |
| FPYBL077W | TCGGTGGAGACAGTTAGCATT |
| RPYBL077W | ACGTACAAATTTTTCTCTACTCGAA |
| FPYHR216W | CCCTGTCACGACAGATGGAA |
| RPYHR216W | CGCCGGTAACAGGGTTTTTG |
| FPYBR263W | TGCCATACCATGTCGACCAC |
| RPYBR263W | CTCGAGTACGCGGAAGTACC |
| pNG158-FP | CTCTAGAACTAGTGGATCCCGAGGGTTCAGGGAAATCC |
| pNG158-RP | TACCGGGCCCCCCCTCGAGGTGTTTAACTCTTGCGTTACTATG |
| FPTRK2LUC | CTAGAACTAGTGGATCCCCCTTCAGAACCGATGCTGGGGTATT |
| RPTRK2LUC | TCTTCCAAGGTGAAAACCATCCCATGCTTCCCCCAAAACTTTGTTG |
| FPIDP2LUC | CTAGAACTAGTGGATCCCCCATGACGACATGGAAGTCGTCAATAG |
| RPIDP2LUC | TCTTCCAAGGTGAAAACCATCCCCAATGCAGCTGCCTCGAACTCTT |
| FPTHI21LUC | CTAGAACTAGTGGATCCCCCTTGATCCTATTACATTATCAATCCTTGCG |
| RPTHI21LUC | TCTTCCAAGGTGAAAACCATCCCTTCATATTCTAGAGCGGCGGTC |
| FPAMD2LUC | CTAGAACTAGTGGATCCCCCGATTTTTTTATGTCAAGCACCTACTTTTA |
| RPAMD2LUC | TCTTCCAAGGTGAAAACCATCCCTATTTTGCTAAAAAGCCAAGACGCAAAT |
| FPPCK1LUC | CTAGAACTAGTGGATCCCCCTCGTTCGTTGTACGTACATTTACAAC |
| RPPCK1LUC | TCTTCCAAGGTGAAAACCATCCCTCGAATTGAGGACCAGCGG |
| FPSAG1LUC | CTAGAACTAGTGGATCCCCCCAAAATGTTGCAGATCTGTGACGA |
| RPSAG1LUC | TCTTCCAAGGTGAAAACCATCCCGAATAGCAGGTACGACAAAAGCA |
| FPFLO5LUC | CTAGAACTAGTGGATCCCCCAAATTCTGTTTTTAATAGCCAGTTCTTTAG |
| RPFLO5LUC | TCTTCCAAGGTGAAAACCATCCCAATAATTGCCAGCAATAAGGACGC |
| FPFLO1LUC | CTAGAACTAGTGGATCCCCCAGTGCATTTATTTAGGTAAGTCTCATTAC |
| RPFLO1LUC | TCTTCCAAGGTGAAAACCATCCCAATAATTGCCAGCAATAAGGACGC |
| FPSNZ1LUC | CTAGAACTAGTGGATCCCCCATACTTTAAGATATGGTTTTTCTTTCTATTTAAC |
| RPSNZ1LUC | TCTTCCAAGGTGAAAACCATCCCCCACCCAATTTCGGAAAGTCTTAC |
| FPADR1LUC | CTAGAACTAGTGGATCCCCCGGCCTCCCCGGAATTTACC |
| RPADR1LUC | TCTTCCAAGGTGAAAACCATCCCACTGTTTCCCTTTAGATGATTTTCC |
| FPFIG2LUC | CTAGAACTAGTGGATCCCCCCAGCAATTATGTGTCTTTGGGCAAC |
| RPFIG2LUC | TCTTCCAAGGTGAAAACCATCCCAATGAAGTTGATCACCATTACCATTAT |

**Supplementary File 1d: Details of *nLUC* reporter genes:**

| gene_ID | Gene name | 5' UTR length (nt) | CDS length (nt) | 3' UTR length (nt) |
| --- | --- | --- | --- | --- |
| *YBL075C* | *SSA3* | 467 | 1950 | 181 |
| *YJR004C* | *SAG1* | 1126 | 1953 | 332 |
| *YKR050W* | *TRK2* | 1261 | 2670 | 268 |
| *YDR242W* | *AMD2* | 408 | 1650 | 242 |
| *YPL258C* | *THI21* | 1450 | 1656 | 191 |
| *YLR174W* | *IDP2* | 519 | 1239 | 197 |
| *YMR096W* | *SNZ1* | 450 | 894 | 275 |
| *YHR211W* | *FLO5* | 2754 | 3228 | 185 |
| *YCR107W* | *AAD3* | 878 | 1092 | 365 |
| *YCR089W* | *FIG2* | 614 | 4830 | 694 |
| *YAR050W* | *FLO1* | 3185 | 4614 | 162 |
| *YDR216W* | *ADR1* | 958 | 3972 | 407 |
| *YKR097W* | *PCK1* | 771 | 1650 | 157 |

**Supplementary File 1e: Codon-optimized *nLUC* coding sequence for yeast (516 nts)**

| Sequence in 5’ to 3’ |
| --- |
| ATGGTTTTCACCTTGGAAGATTTTGTTGGTGATTGGAGACAAACTGCTGGTTACAATTTGGATCAAGTTTTGGAACAAGGTGGTGTTTCTTCTTTGTTTCAAAATTTGGGTGTTTCTGTTACCCCAATTCAAAGAATTGTTTTGTCTGGTGAAAATGGTTTGAAGATTGATATTCATGTTATTATTCCATACGAAGGTTTGTCTGGTGATCAAATGGGTCAAATTGAAAAGATTTTCAAGGTTGTTTACCCAGTTGATGATCATCATTTTAAGGTTATTTTGCATTACGGTACTTTGGTTATTGATGGTGTTACTCCAAATATGATTGATTACTTTGGTAGACCATACGAAGGTATTGCTGTTTTTGATGGTAAGAAGATTACTGTTACTGGTACTTTGTGGAATGGTAATAAGATTATTGATGAAAGATTGATTAATCCAGATGGTTCTTTGTTGTTCAGAGTTACTATTAATGGTGTTACTGGTTGGAGATTGTGTGAAAGAATTTTGGCTTAA |

**Supplementary File 1 References**

Charenton C, Gaudon-Plesse C, Fourati Z, Taverniti V, Back R, Kolesnikova O, Seraphin B, Graille M. 2017. A unique surface on Pat1 C-terminal domain directly interacts with Dcp2 decapping enzyme and Xrn1 5'-3' mRNA exonuclease in yeast. *Proc Natl Acad Sci U S A* **114**: E9493-E9501.

Gietz RD, Sugino A. 1988. New yeast-Escherichia coli shuttle vectors constructed with in vitro mutagenized yeast genes lacking six-base pair restriction sites. *Gene* **74**: 527-534.

He F, Jacobson A. 2001. Upf1p, Nmd2p, and Upf3p regulate the decapping and exonucleolytic degradation of both nonsense-containing mRNAs and wild-type mRNAs. *Mol Cell Biol* **21**: 1515-1530.

He F, Li X, Spatrick P, Casillo R, Dong S, Jacobson A. 2003. Genome-wide analysis of mRNAs regulated by the nonsense-mediated and 5' to 3' mRNA decay pathways in yeast. *Mol Cell* **12**: 1439-1452.

Sikorski RS, Hieter P. 1989. A system of shuttle vectors and yeast host strains designed for efficient manipulation of DNA in *Saccharomyces cerevisiae*. *Genetics* **122**: 19-27.

Zeidan Q, He F, Zhang F, Zhang H, Jacobson A, Hinnebusch AG. 2018. Conserved mRNA-granule component Scd6 targets Dhh1 to repress translation initiation and activates Dcp2-mediated mRNA decay in vivo. *PLoS Genet* **14**: e1007806.
